# G Protein-Coupling of Adhesion GPCRs ADGRE2/EMR2 and ADGRE5/CD97, and Activation of G Protein Signalling by an Anti-EMR2 Antibody

**DOI:** 10.1038/s41598-020-57989-6

**Published:** 2020-01-22

**Authors:** Nisha Bhudia, Sapna Desai, Natalie King, Nicolas Ancellin, Didier Grillot, Ashley A. Barnes, Simon J. Dowell

**Affiliations:** 1 Medicinal Science and Technology, GlaxoSmithKline, Stevenage, UK; 2 Excelya Clinical Research, Boulogne-Billancourt, France; 3 Oncodesign, Villebon-Sur-Yvette, France; 4 Censo Biotechnologies Ltd., Babraham, UK

**Keywords:** Biochemistry, Biological techniques, Cancer, Cell biology, Drug discovery, Immunology

## Abstract

The experimental evidence that Adhesion G Protein-Coupled Receptors (aGPCRs) functionally couple to heterotrimeric G proteins has been emerging in incremental steps, but attributing biological significance to their G protein signalling function still presents a major challenge. Here, utilising activated truncated forms of the receptors, we show that ADGRE2/EMR2 and ADGRE5/CD97 are G protein-coupled in a variety of recombinant systems. In a yeast-based assay, where heterologous GPCRs are coupled to chimeric G proteins, EMR2 showed broad G protein-coupling, whereas CD97 coupled more specifically to G_α12_, G_α13_, G_α14_ and G_αz_ chimeras. Both receptors induced pertussis-toxin (PTX) insensitive inhibition of cyclic AMP (cAMP) levels in mammalian cells, suggesting coupling to G_αz_. EMR2 was shown to signal via G_α16_, and via a G_α16_/G_αz_ chimera, to stimulate IP_1_ accumulation. Finally, using an NFAT reporter assay, we identified a polyclonal antibody that activates EMR2 G protein signalling *in vitro*. Our results highlight the potential for the development of soluble agonists to understand further the biological effects and therapeutic opportunities for ADGRE receptor-mediated G protein signalling.

## Introduction

The Adhesion G Protein-Coupled Receptors (aGPCRs) constitute an evolutionarily ancient membrane protein family with emerging roles in many important biological processes (for reviews see^1–5^). The receptors each contain a 7-transmembrane (7-TM) domain with phylogeny suggesting ancestry to the ‘Family B’ (Secretin receptor family; also known as Class B) G Protein-Coupled Receptors (GPCRs). However, aGPCRs are distinguished by their large amino-terminal regions that typically contain multiple modular motifs such as EGF (Epidermal Growth Factor-like), cadherin and immunoglobulin domains as well as novel lineage-specific structures. While these are generally thought to mediate inter-cellular ‘adhesion’ interactions, various examples suggest separable roles for the extracellular domain (ECD) and 7-TM regions^6,7^. This complexity, apart from the sheer size of some of the receptors, underlies some of the challenges of studying aGPCRs.

As the known interacting partners of aGPCRs are usually tethered to other cells, their identification and characterisation have been difficult. Of those identified, including CD55 for CD97^8^, transglutaminase II (TGII) for GPR56^9^ and chondroitin sulphate B (dermatan sulphate) for EMR2^10^, few had measurable effects on G protein signalling *in vitro* and it is not clear whether these partners can be considered ‘ligands’, as understood for the better characterised Family A (Rhodopsin-like), Family B (Secretin receptor family) or Family C (Metabotropic glutamate family) GPCRs, that modulate G protein signalling pathways in response to the binding of soluble activators. Indeed, only recently has evidence become compelling of aGPCR association with G protein alpha subunits (reviewed in Langenhan *et al*.^11^), changes in second messenger levels^11^, and GTP turnover in membranes from cells expressing aGPCRs in combination with specific G_α_ subunits^12^.

A defining feature of aGPCRs is the GPCR Autoproteolysis-INducing (GAIN) domain linking the amino-terminal structure to the 7-TM region^13^. Most aGPCRs undergo autocatalytic cleavage here, at the GPCR Proteolysis Site (GPS), into N-terminal (NTF) and C-terminal (CTF) fragments connected by a disulphide bridge. While exogenous pharmacological tools have been elusive, a breakthrough was the finding that removal of the NTF appears to activate many aGPCRs (^11,14–18^), the mechanism for this being the subject of extensive discussion (reviewed in Purcell *et al*.^14^), with evidence for a tethered agonist (sometimes named the *‘Stachel’*) in some aGPCRs, that becomes exposed upon removal of the NTF^12,15^. Several exceptions appear to rule out the *Stachel* as a universal mechanism (reviewed in Purcell *et al*.^14^). Moreover, the biological significance of G protein signalling for most aGPCRs remains undetermined.

The aGPCRs are further classified into sub-families based on the nature of their N-terminal domains. One such sub-family, the ADGRE family (EMR1, 2, 3, 4 and CD97; also known as the ‘EGF-7TM’ sub-family) is characterised by the presence of several tandem EGF domains in their NTFs^16^. EMR2 (EGF-Like Module-Containing Mucin-Like Hormone Receptor-Like 2) and CD97 (Cluster of Differentiation 97) have similar alternatively spliced gene structures^17–19^ each encoding five EGF domains, with 97% identity in their amino-terminal regions, but the receptors differ in several ways. Only CD97 has high affinity for the complement regulatory protein, Decay Accelerating Factor (DAF; CD55)^1^, mediated by EGF domains 1 and 2 which differ in just three amino acids from those in EMR2. In both receptors the fourth EGF domain binds chondroitin sulphate^10,20,21^ which, in EMR2, may recruit macrophages in the inflamed synovium of rheumatoid arthritis patients^20^; however, the prevailing CD97 variant on leukocytes contains only EGF domains 1, 2 and 5^22^, indicating that the receptors have non-redundant functions. CD97 also binds Thy-1^23^ and α_5_β_1_ and α_v_β_3_ integrins^24^. The receptors also differ in their tissue distribution: where CD97 is distributed more broadly and found on all types of haematopoietic cells as well as on smooth muscle cells^19,25–27^, EMR2 is restricted to myeloid cells including monocytes, macrophages, dendritic cells and granulocytes^28^. EMR2 expression is highly regulated during monocyte/macrophage differentiation^29^, and a missense mutation in EMR2 is linked to vibratory urticaria^30^.

Details of ADGRE G protein signalling have been slow to emerge. CD97 induced G_α12_-dependent activation of a SRE-Luciferase reporter gene, enhanced by removal of the CD97 NTF^31^. CD97 signalling, linked to interactions with LPAR1, is attributed to cause platelet activation during tumour metastasis^32^. A role for GRK6 in CD97 signalling desensitisation (likely via β-arrestin 1 recruitment) has been reported^33^. EMR2 was shown to induce inositol phosphate accumulation via the rodent G protein G_α15_, in HEK 293 cells^34^, but no coupling was seen to chimeric G proteins G_qi5_, G_qi9_, G_qo3_, G_qo5_ or G_α12_. A selective monoclonal Anti-EMR2 antibody (mAb), 2A1^28^, that binds to EMR2’s GAIN domain, induced the potentiation of polymorphonucleocyte (PMN) migration, degranulation, and the production of Reactive Oxygen Species (ROS) towards inflammatory stimuli, and EMR2 truncation demonstrated that the 7-TM region is critical for cell migration^35^. By inference, 2A1 is considered an activating antibody of EMR2. 2A1’s effect on EMR2 signalling requires cross-linking or immobilisation, and gene knockdown studies indicate that EMR2’s coupling to PLC-β activation is via G_α16_^36^.

The tissue expression and biology of EMR2 and CD97 suggest possible roles in inflammatory/immune disorders, and a key question is whether modulation of their G protein signalling could provide a tractable therapeutic opportunity, as precedented for medicines that act on Family A GPCRs. We set out to confirm G protein coupling of EMR2 and CD97 in various systems including yeast and mammalian cell signalling assays. We identify direct G protein-coupling of EMR2 and CD97 and, for the first time, a soluble activating antibody that stimulates G protein-coupling of EMR2 in mammalian cells, that could be a useful tool to probe the biological effects of EMR2 signalling.

## Results

### Demonstration of G protein-coupling of EMR2 and CD97 in a yeast reporter assay

**Figure 1:**
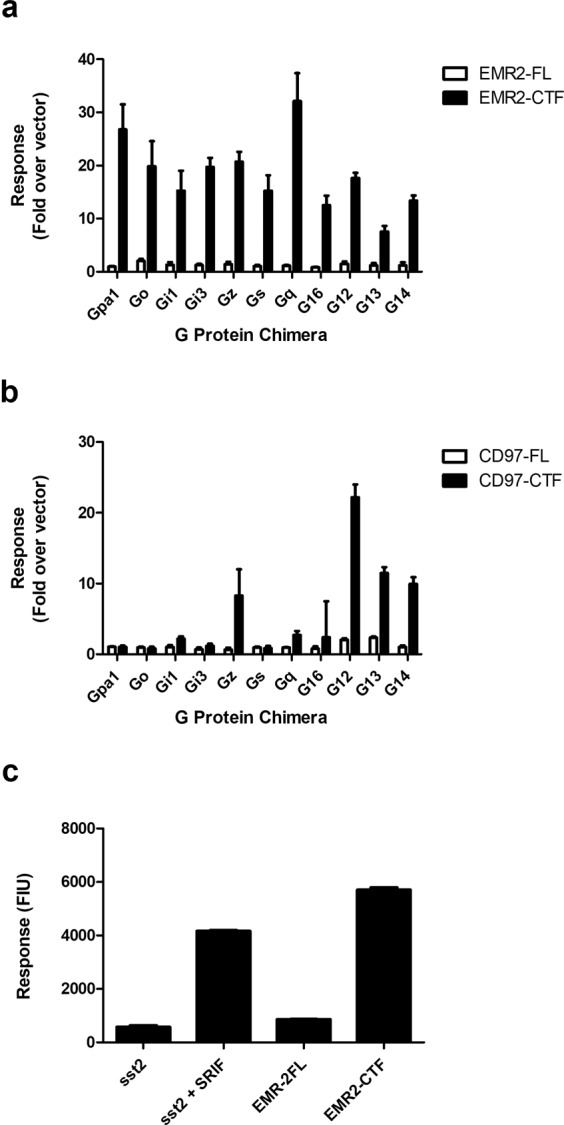
EMR2 and CD97 stimulate G protein coupling in a yeast-based reporter assay. Constructs expressing full-length (-FL) or truncated (-CTF) forms of EMR2 (**a**) and CD97 (**b**) were transformed into yeast strains expressing yeast Gpa1p or chimeric Gpa1/G protein alpha subunits corresponding to mammalian G_α_ sequences as designated on the x axis. Fluorescence Intensity Units (ex. 485 nm/em. 535 nm) report receptor-stimulated cell growth leading to the production of fluorescein. Data are presented as fold increase over a vector control. (**c**) Responses induced by EMR2-FL and EMR2-CTF were compared with human somatostatin receptor unstimulated (sst_2_) or stimulated (sst_2_ + SRIF) with a saturating concentration (1 μM) of somatostatin (SRIF-14). All data are plotted as the mean + range of four independent isolates, two replicates of each.

We initially investigated G protein-coupling of EMR2 in a yeast reporter assay. In this system, GPCR activation is coupled to a growth response via modified yeast G protein alpha (Gpa1p) subunits in which the C-terminal five amino acids are replaced with corresponding mammalian G_α_ sequences (see^37,38^). An attraction of this assay is that the engineered yeast have no endogenous GPCRs capable of activating the reporter. Furthermore, since the signal is transduced by the G_βγ_ particle, coupling via different G protein alpha species can be compared using the same readout. In the absence of known activating ligands, constructs encoding full length EMR2 (EMR2-FL; five EGF variant) and the EMR2 C-terminal fragment (EMR2-CTF) were transformed into eleven yeast strains containing different chimeric G_α_ subunits, to determine whether removal of the NTF might activate EMR2. Compared with a vector control, full length EMR2 showed small levels of constitutive activity (Fig. 1a), similar to the constitutive activity often seen for G protein-coupled receptors in the absence of an agonist^38^. However, strikingly, the EMR2-CTF stimulated significant activation in all the strains (Fig. 1a), indicating an ability to couple to chimeric G proteins across all the G_αi/o_, G_αs_, G_αq_ and G_α12/13_ families. CD97 (full length EGF1, 2, 5 variant, ‘CD97-FL’) was also tested for activity in the yeast system, and as with EMR2, the truncated CD97-CTF form stimulated high levels of pathway activation compared with CD97-FL (Fig. 1b). However, CD97’s G protein coupling specificity was more restricted than EMR2’s, with the strongest activity seen in the G_αz_, G_α12_, G_α13_ and G_α14_ strains. Very little activity was seen in strains expressing G_αi/o_ and G_αs_ chimeras. The level of EMR2 activation was slightly higher than that of the human somatostatin receptor sst_2_ maximally stimulated with the agonist SRIF-14 (Fig. 1c). These experiments confirm that EMR2 and CD97 can couple directly to heterotrimeric G proteins and support the model in which removal of the NTF activates G protein signalling by aGPCRs.

### EMR2 and CD97 inhibit cAMP accumulation in a pertussis toxin-insensitive manner in mammalian cells

**Figure 2:**
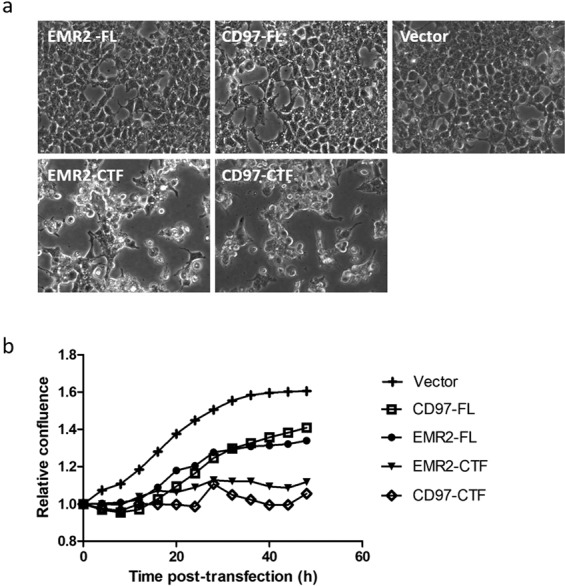
Activated forms of EMR2 and CD97 affect HEK 293 T cell growth. (**a**) Cell images visualised using IncuCyte illustrate the effects on cell monolayer confluence and morphology at 48 hours after transfection with EMR2-FL, EMR2-CTF, CD97-FL and CD97-CTF constructs or a vector control (Vector; pcDNA3) at 0.4 μg DNA/cm^2^ cells. Each panel represents a separate, independent image of cells transfected with the respective constructs (**b**) Quantification of cell growth, presented as Relative Confluence, measured as level of confluence, at the time (hours) indicated after transfection of the constructs (at 0.4 μg DNA/cm^2^ cells), divided by the confluence at t = 0.

**Figure 3:**
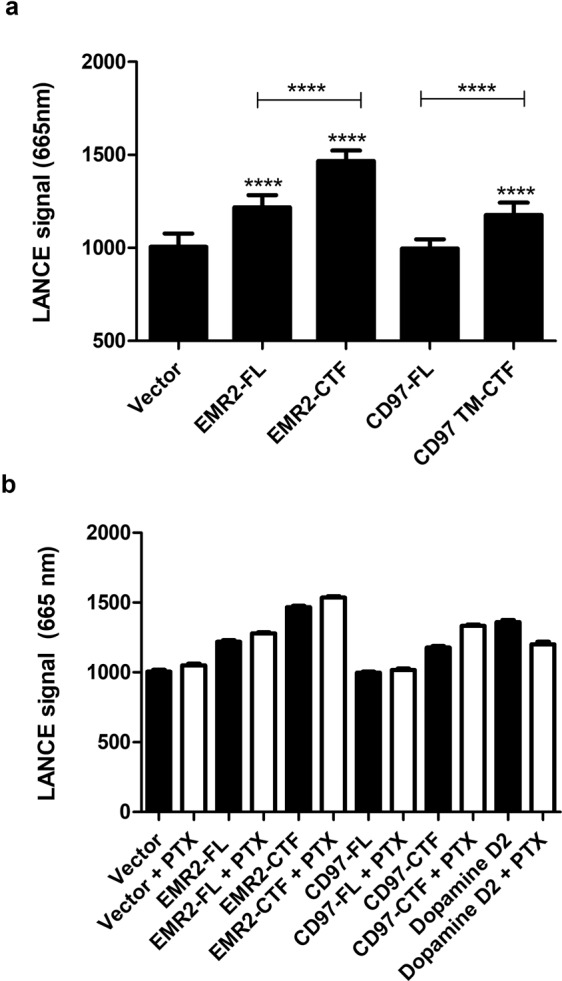
EMR2-FL, EMR2-CTF and CD97-CTF reduce intracellular cAMP levels. Vector (pcDNA3), EMR2, CD97 or Dopamine D2R (control) constructs (each at 0.1 μg DNA/m^2^ cells) were transfected into HEK 293 T cells and the effects on cAMP measured using the LANCE TR-FRET assay (Perkin-Elmer). In panel (a) cells were pre-stimulated with an EC_80_ of forskolin (200–800 nM). Inhibition of cAMP accumulation is indicated as an increase in TR-FRET signal (fluorescence intensity at 665 nm). In panel (b) cells were pre-stimulated with forskolin plus overnight treatment with no PTX (black bars) or 100 ng/ml PTX (clear bars). PTX did not affect the cAMP inhibition elicited by EMR2 or CD97 but reduced the cAMP inhibition elicited by D2R. Data are the mean ± SEM of minimum of three independent experiments. Statistical significance was measured using unpaired *t*-test (*p < 0.05).

Using the principle established in our yeast experiments, we investigated whether the ‘activated’ EMR2-CTF or CD97-CTF might stimulate G protein coupling transiently when transfected into mammalian cells. Introduction of EMR2 constructs into HEK 293 T cells had significant effects on cell growth (Fig. 2a), with full length EMR2 (EMR2-FL; DNA concentration at 0.4 μg/cm^2^ of cells) reducing the growth rate by approximately 50% (Fig. 2b), and EMR2-CTF arresting growth almost completely, suggesting that EMR2 was being expressed. This effect was titratable, with marginal effects on cell growth at 0.1 μg DNA/cm^2^ cells, and intermediate effects at 0.2 μg DNA/cm^2^ cells (Supplementary Fig. S1a–c). Effects of the aGPCRs on cAMP accumulation were investigated using the LANCE cAMP assay (Perkin-Elmer, Waltham, MA, USA). In this system, time-resolved fluorescence resonance energy transfer (TR-FRET), between a Europium-labelled cAMP chelate complex and Alexa-Fluor 647-labelled specific cAMP antibodies, is disrupted by the generation of cAMP in the cell. Inhibition of cAMP accumulation is seen as a positive fluorescence intensity signal at 665 nm. In the LANCE assay, no significant elevation of cAMP was seen upon expression of EMR2 (Supplementary Fig. S2a). However, in cells treated with an EC_80_ of forskolin, an activator of adenylyl cyclase, intracellular cAMP levels were significantly reduced in cells transduced with EMR2-FL, and even more significantly with EMR2-CTF (Fig. 3a), relative to cells transduced with a control vector. The inhibition of cAMP occurred at all DNA concentrations tested (0.1, 0.2 and 0.4 μg DNA/cm^2^ cells; Supplementary Fig. S2a), for both EMR2-FL and EMR2-CTF constructs, even though the effects on cell growth were marginal at 0.1 μg DNA/cm^2^ cells. Interestingly, the apparent inhibition of cAMP accumulation was not blocked by the action of pertussis toxin (PTX), an inhibitor of G_αi_ G proteins^39^, whereas the cAMP reduction caused by activation of a control GPCR, Dopamine D2R, was reversed by PTX (Fig. 3b). CD97 constructs also caused negative effects on HEK 293 T cell growth (Fig. 2a), with CD97-FL (0.4 μg DNA/cm^2^ cells) reducing the growth rate by approximately 50% and CD97-CTF arresting growth almost completely (Fig. 2b). As for EMR2 the effects were titratable, with increasing DNA concentrations giving more pronounced effects (Supplementary Fig. S1a). In LANCE cAMP experiments, expression of CD97-FL had no significant effect on cAMP levels (Fig. 3a; Supplementary Fig. S2b); however, CD97-CTF significantly inhibited cAMP accumulation in forskolin-treated cells (Fig. 3a), and effects were measurable at the lowest concentration of DNA tested (0.1 μg DNA/cm^2^ cells; Supplementary Fig. S2b), where there was marginal effect on cell growth. This inhibition of cAMP accumulation was not reversed by the addition of PTX (Fig. 3b).

### EMR2 stimulates IP_1_ accumulation via G_α16_ in mammalian cells

**Figure 4:**
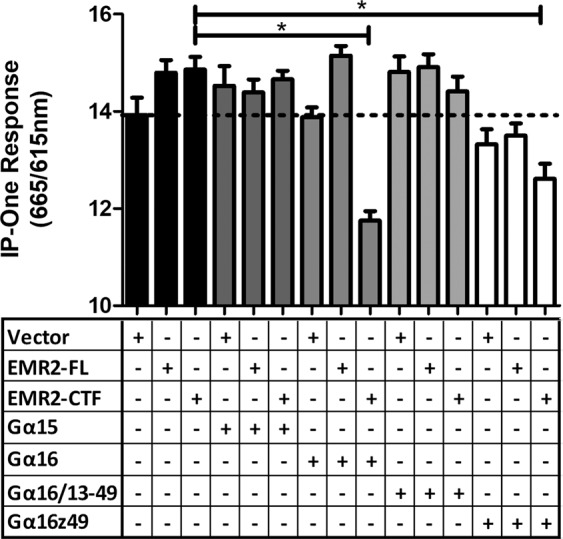
Activation of Inositol phosphate production by EMR2. EMR2-FL, EMR2-CTF and vector control constructs were transfected into HEK 293 T cells in combination with different G protein alpha subunits, as indicated, and tested in the IP-One HTRF assay (Cisbio). A reduction in the ratio (665 nm/615 nm) indicates an accumulation of inositol monophosphate, a downstream metabolite of IP_3_ induced by activation of a phospholipase C cascade. Data represent mean ± SD of 16 replicates for each condition. Statistical significance was measured using unpaired *t*-test (*p < 0.05).

In another attempt to confirm G protein coupling in mammalian cells, we investigated the potential of EMR2 to stimulate inositol monophosphate (IP; IP_1_) production using the IP-One assay (Cisbio, Codolet, France). IP_1_ is an indicator of the activation of phospholipase C (PLC) by the G_αq_ class of G proteins, which includes G_α16_^40^. In this assay, a homogenous time-resolved FRET (HTRF) signal, generated by an interaction between anti-IP_1_ Cryptate (donor) and d2-labelled IP_1_ (acceptor), is disrupted by the the presence of free IP_1_ in the cells. Accumulation of IP_1_ as a result of GPCR signalling is indicated as a decrease in the fluorescence ratio (665 nm/615 nm). HEK 293 T cells were transiently transfected with EMR2-FL or EMR-CTF constructs alongside a vector control (pcDNA3). Neither EMR2-FL nor EMR2-CTF alone stimulated any measurable IP_1_ accumulation, nor was any IP_1_ accumulation seen when the G protein alpha subunit G_α15_ was co-transfected (Fig. 4). However, when co-transfected with G_α16_, EMR2-CTF gave a striking response indicative of IP_1_ accumulation. These data give direct evidence of signalling of the EMR2-CTF via G_α16_ in mammalian cells. We saw no significant IP_1_ accumulation with EMR2-CTF in combination with a version of G_α16_ in which the C-terminal region was replaced with sequence from G_α13_ (G_16/13–49_). However, there was a significant stimulation of IP_1_ levels when EMR2-CTF was co-expressed with G_α16_ incorporating the C-terminal 49 residues of G_αz_ (G_16z49_).

### EMR2 stimulates an NFAT-Luciferase reporter

**Figure 5:**
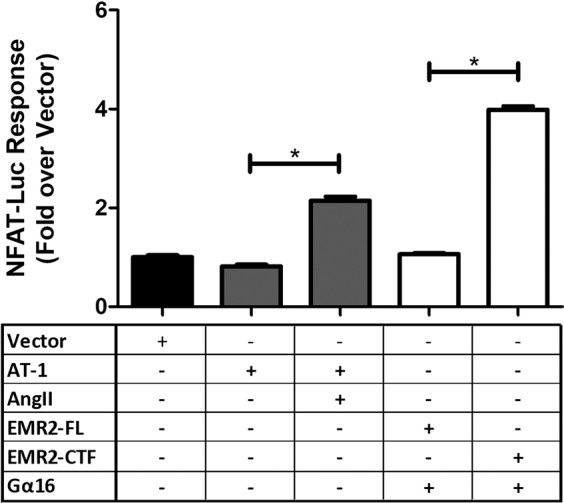
Stimulation of the NFAT-Luciferase reporter by EMR2. Data indicate NFAT activation when vector, EMR2-FL EMR2-CTF or AT-1 constructs were transfected into HEK 293 T cells containing an NFAT-Luciferase reporter. The EMR2 constructs were co-transfected with a G_α16_ expression construct.). Data (RLU; Relative Light Units) represent mean ± SD of a minimum of 6 replicates of each condition. Statistical significance was measured using unpaired *t*-test (*p < 0.05).

We also investigated EMR2 signalling in HEK 293 T cells, using a luciferase reporter gene linked to the NFAT response element. NFAT (Nuclear Factor of Activated T cells) transcription factors are important in the immune response and are regulated by the influx of calcium^41^. NFAT-based reporters generally indicate the activity of G proteins that induce calcium release, such as G_αq_ and G_α16_. As a control in this assay, the Angiotensin AT-1 receptor stimulated significant NFAT-Luciferase activity when challenged with 25 μM AngII agonist (Fig. 5). Full length EMR2 transiently co-transfected with G_α16_ in this cell line generated no significant reporter activity; however, EMR2-CTF co-transfected with G_α16_ stimulated a significant increase in NFAT-Luciferase activity, considerably higher than the AT-1 response (Fig. 5). No luciferase activity was stimulated either by EMR2-FL or EMR2-CTF using a control reporter that lacked the NFAT response element (pGL4.14) (Supplementary Fig. S3). These results confirmed G protein coupling of EMR2 in an alternative mammalian cell-based assay system.

### Identification of an activating antibody to EMR2

**Figure 6:**
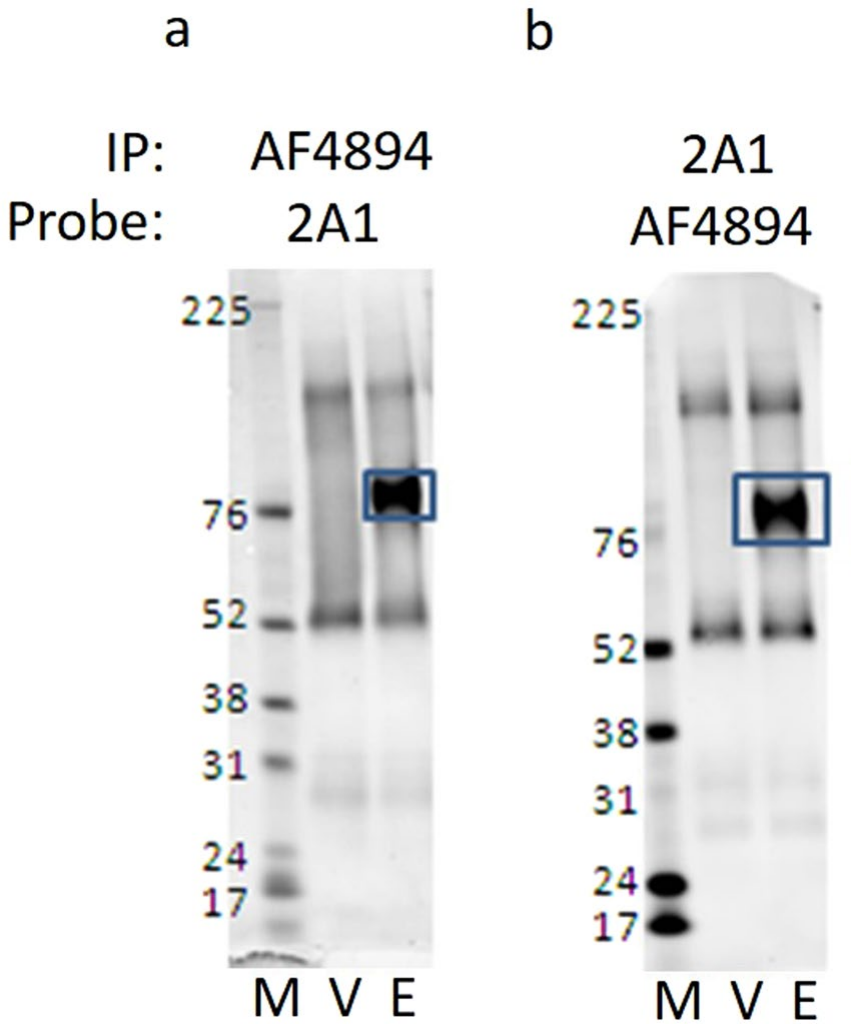
Detection of EMR2 by commercial antibodies. HEK 293 T cells were transfected with a control BacMam virus (V) or with BacMam virus engineered to produce EMR2 (E). Protein size markers are indicated (M). Immunoprecipitation (IP) was carried out from cell lysates using R&D Systems anti-EMR2 pAb AF4894 (panel a), or Serotec mAb 2A1 (panel b) and immunoprecipitates were subjected to electrophoresis on the same gel. Following transfer, the membrane was divided into two strips which were probed with (**a**) 2A1, or (**b**) AF4894, respectively. These strips are presented in their entirety side by side as panels a and b. The specific EMR2 band is boxed in each case.

**Figure 7:**
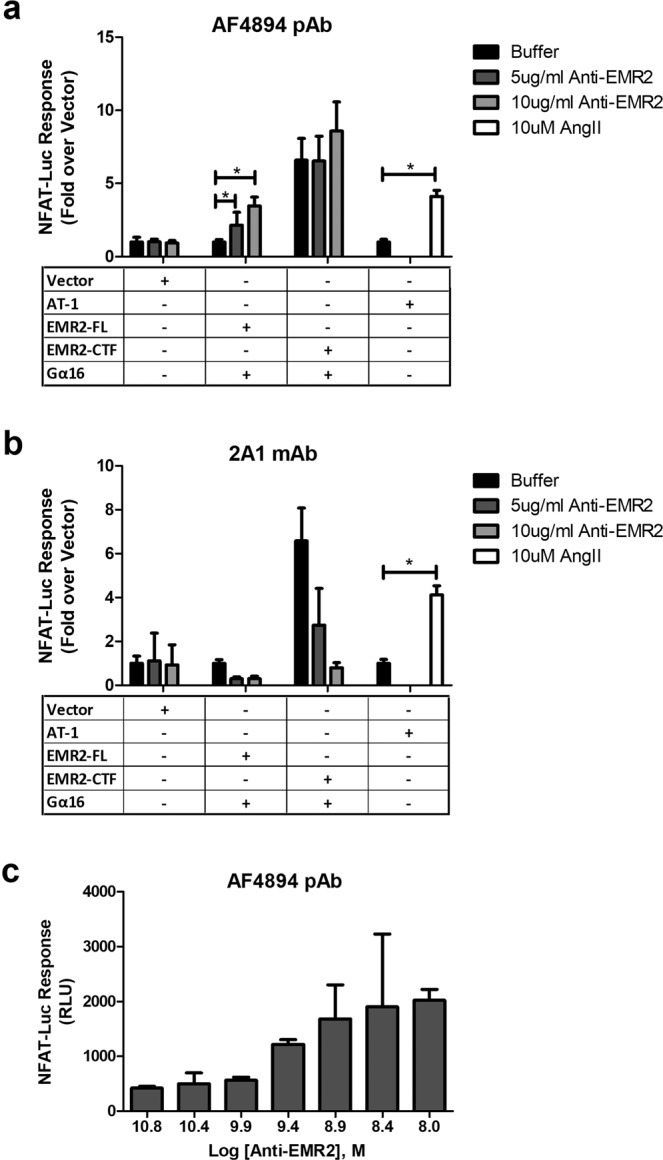
Activation of EMR2 by an antibody. NFAT-Luciferase reporter (NFAT-Luc) responses were measured following transient co-transfection of Vector, EMR2-FL, EMR2-CTF or AT-1 constructs with an NFAT-Luciferase reporter construct into HEK 293 T cells, and treatment with EMR2 antibodies (5–10 mg/ml) or Ang II (25 μM) as indicated: (panel a) pAb R&D Systems AF4894, (panel b) Serotec mAb, 2A1. Data were normalised as fold effect relative to vector control. (Panel c) illustrates the NFAT-Luciferase activity (RLU, Relative Light Units) in EMR2-FL transfected cells treated with pAb EMR2 (R&D Systems), at the indicated antibody concentrations.

Having established methods for detecting G protein coupling of EMR2 and CD97, we were interested to know whether any available tools might have effects on aGPCR-mediated G protein signalling. Exogenously applied dermatan sulphate, or EMR2-derived *Stachel* peptides (gift of Ines Liebscher, University of Leipzig), failed to stimulate agonist responses, although the peptides were insoluble. Soluble CD55 protein failed to activate CD97 in our assays. We acquired the commercially available mAb known as 2A1 (Serotec) and a polyclonal sheep anti-human EMR2 antibody (pAb AF4894) from R&D Systems, both raised to the external region of EMR2. Both antibodies were effective in immunoprecipitation of EMR2 from HEK 293 T cells transduced with modified baculoviruses (BacMams) engineered to express these aGPCRs in mammalian cells, and both were active in detecting EMR2 by Western blotting (Fig. 6a,b). Both antibodies were selective for EMR2 over CD97. 2A1 was of interest, being described as an activating antibody based on its effects on cytokine release^35^. Using the NFAT-Luciferase assay in transiently transfected HEK 293 T cells, as before, in the absence of antibody, EMR2-CTF co-transfected with G_α16_ stimulated significant luciferase activity compared with a vector (pcDNA3) or EMR2-FL (Fig. 7a,b, black bars). The pAb AF4894 had no effect on the basal NFAT-Luciferase activity level in vector transfected cells (Fig. 7a). In cells transfected with truncated EMR2-CTF, which were already maximally stimulated, AF4894 had no significant additional effect, either positive or negative. However, in the cells transfected with full length EMR2, AF4894 stimulated NFAT-Luciferase activity, suggestive of G protein activation (Fig. 7a). When EMR2-FL transfected cells were treated with increasing amounts of the 2A1 mAb, there was no antibody-mediated stimulation of the reporter but a decrease in the reporter activity was seen (Fig. 7b). However, decreases were also seen in the NFAT-Luciferase response in cells co-transfected with EMR2-CTF. Given that the antibody does not bind to the portion of EMR2 expressed by this construct, the results suggest that the effect of 2A1 in this assay was unspecific. The activity of the AF4894 pAb at EMR2-FL was titratable over a concentration range approximately 0.1–10 nM (Fig. 7c).

## Discussion

Despite the relatively early discovery of EMR2 and CD97 within the aGPCR family, evidence of their G protein-coupling has been limited. EMR2 was reported to couple to rodent G_α15_ in a recombinant assay^34^, and to mediate inflammatory responses through G_α16_ activation^36^. CD97 coupling via G_α12/13_ has been indicated, involving heterodimerization with the lysophosphatidic acid receptor LPAR1^31^; and the PTX-sensitive lysophosphatidylethanolamine (LPE) response of LPAR1 in MDA-MB-231 cells requires CD97^42^. GRK6-mediated desensitisation of CD97^33^ further indicates its participation in classical GPCR signalling mechanisms. In view of the complexity of interpreting signalling pathways from primary cell data, we were interested to identify defined recombinant systems to investigate aGPCR signalling mechanisms under well-controlled conditions. The yeast reporter assay is a novel approach in the aGPCR field, that has been useful previously for demonstrating G protein coupling of mammalian GPCRs due to its robustness and the ability to test individual components, for instance different G protein chimeras, in a minimal non-mammalian cell background^38^. The appeal of the system is that the host *Saccharomyces cerevisiae* contains only one endogenous GPCR capable of activating the pheromone-response pathway, and this has been deleted in the engineered strains. The endogenous yeast G_α_ subunit has also been deleted, so receptor coupling to the downstream pathway can only be achieved through the use of added G protein alpha subunits. The chimeric G proteins used here are composed primarily of the yeast G_α_, Gpa1p, enabling compatibility with the yeast G_βγ_ particle, but with the C-terminal five amino acids replaced by corresponding mammalian G_α_ sequences, where the predominant receptor selectivity determinants reside. When the G_α_ is activated, the G_βγ_ particle is released to activate the downstream pathway, leading to induction of a *FUS1-HIS3* reporter gene and the production of histidine. Yeast growth in histidine-deficient media is seen as a direct measure of GPCR activation. The fact that GPCRs exhibit selectivity for individual G_α_ chimeras is confirmation that they are signalling through this defined pathway and not through another unexplained mechanism.

In the absence of ligands, we used the principle that truncating the aGPCRs might activate the receptors, to investigate G protein activation by EMR2 and CD97 in the recombinant systems tested. The large responses elicited by truncated EMR2 (EMR2-CTF) and CD97 (CD97-CTF) in the yeast assays provide incontrovertible evidence of direct G protein-coupling of both receptors. Although we did not investigate differences in expression levels between full-length and truncated forms, the signal from both truncated receptors exceeded that of the Family A GPCR sst_2_ fully agonised with SRIF-14, consistent with the model that truncation activates these aGPCRs. EMR2 signalling was detectable with all eleven G protein species tested. It is not uncommon for mammalian GPCRs to couple to several different chimeric G proteins in yeast^38^, though we infer that EMR2 signalling is particularly strong. Usually, differences in ligand potency between strains indicate the physiological relevance of the G proteins^38^, which is hard to assign for EMR2 in the absence of a ligand given the broad range of activity seen. CD97, however, had a more selective coupling profile, with a clear preference for G_α12_ and G_α13_, G_α14_ and G_αz_ chimeras. Given the similarity of yeast Gpa1p to mammalian G_αi_ G proteins^43,44^, and our own experience that G_αi_-derived chimeras tend to be the most generic (Ref.^38^ and unpublished data), it is significant that CD97 failed to couple to G_αi/o_ chimeras in this system.

Our attempts to detect EMR2 and CD97 activation in stable mammalian cell lines failed, but transient transfection yielded unambiguous signalling. CD97 and EMR2, both full-length and truncated, had marked effects on cell growth and morphology. G protein activation assays were thus always normalised to the viable cell number, whereupon effects on cAMP levels were observed, with both receptors causing statistically significant inhibition of cAMP accumulation. EMR2’s activity was somewhat higher than CD97’s, with full length EMR2 inhibiting cAMP accumulation, whereas only CD97-CTF caused this effect. Failure of PTX to reverse these effects suggests that cAMP inhibition was not mediated by G_αi_ or G_αo_, but possibly by G_αz_, a G protein that inhibits cAMP accumulation but is insensitive to PTX^45,46^; for CD97, this is consistent with the yeast G protein coupling profile. Therefore, CD97 coupling to PTX-sensitive G_αi/o_ G proteins previously discussed^42^ might be a function of its heterodimerisation with LPAR1 in some mammalian cells.

Further confirmation of EMR2 G protein coupling in mammalian cells was provided by IP_1_ assays, in which EMR2-CTF mediated IP_1_ accumulation was dependent the presence of G_α16_, in keeping with the biological pathway data of I. *et al*.^36^. In our experiment there was no coupling to G_α15_, as seen by Gupte *et al*.^34^, but we saw coupling through a G_α16z49_ chimera, supporting the G_αz_ coupling hypothesis suggested from the cAMP assay data. The NFAT reporter confirmed G_α16_-mediated activation, specifically, by EMR2-CTF. A positive control receptor, the G_αq_-coupled Family A GPCR AT1, was able to give a robust NFAT reporter response in the absence of exogenously added G_α_ subunits, which we take as evidence for the presence of endogenous G_αq_ in the HEK 293 T host cell line. By inference, EMR2 does not couple via G_αq_ in the mammalian cells, as no signalling was seen in the absence of added G protein. We were unable to obtain reproducible IP_1_ or NFAT reporter responses from CD97, therefore the combined data from yeast, cAMP studies and literature support the more restricted G protein-coupling of CD97 to G_α12/13_ and G_αz_.

Being more robust in our hands than the IP_1_ assay, the NFAT-luciferase assay allowed the evaluation of commercially available antibodies. It was surprising that full length EMR2 was activated by the R&D Systems polyclonal antibody AF4894 but not the monoclonal antibody 2A1, given the comprehensive reports of 2A1’s biological effects. Published reports indicate that 2A1 needs to be immobilised to effect cytokine release. In our experiments, 2A1 was immobilised as described^35,36^, but experiments performed under various conditions failed to elicit any specific G protein signalling. We did also observe inhibitory effects mediated by 2A1 on control assays; therefore, for technical reasons we cannot draw conclusions on 2A1’s ability to activate EMR2 G protein signalling from these systems.

AF4894 was raised against the N-terminal region of EMR2 (Gln24-Gln 478); being a polyclonal antibody, the precise epitope of the active species is hard to define. We were not able to determine whether AF4894 physically dissociates the EMR2 NTF from the CTF, or whether receptor activation is due to conformational effects on the extracellular domain without dissociation. There remains a remote possibility that binding of the antibody to the NTF somehow causes a disruption of the transmembrane region that is not linked to the normal mechanism of receptor activation. Tools such as these antibodies will be useful to further validate the *Stachel* hypothesis, and to elucidate mechanisms of aGPCR activation more comprehensively. 2A1 was also raised against the EMR2 extracellular region and is thought to bind within the GAIN domain^28^. We were not able to demonstrate whether 2A1 competed for the activity of AF4894. However, differences in the binding sites of the antibodies may account for differences in function, and further detailing of this will be useful to dissect the mechanisms.

The G protein-couplings of EMR2 and CD97 described here agree with data from more biologically relevant systems. For EMR2, G_α16_ coupling is consistent with the expression of both receptor and G protein in immune cells, where EMR2 activation of G_α16_ is linked to macrophage-like differentiation of THP-1 cells to mediate inflammatory responses^36^. For CD97, transfection into COS-7 cells induced a SRE-Luciferase reporter through G_α12/13_-mediated Rho activation, and this has been linked to CD97’s role in cancer invasion and metastasis. The ubiquitous expression of G_α12/13_ is consistent with CD97’s potentially broader roles in myeloid, lymphoid, epithelial, muscle and other cell types. The identification of G_αz_ as a potential G protein partner for both CD97 and EMR2 is of uncertain significance. G_αz_’s tissue distribution may suggest a neuronal function^47^, with roles indicated in immune cells and during development, therefore G_αz_ coupling of EMR2 and CD97 may have a biological relevance here. Given the close sequence identity of EMR2 and CD97, their different G protein-coupling profiles represent another factor in the divergent evolution of the receptors. Combined with the differences in tissue distribution, splice variance, and the different G protein complements in the respective cells types where they are expressed, CD97 and EMR2 are positioned for distinct differentiated functions that can be further probed through the development of specific pharmacological tools.

A principal interest of our work is the development of recombinant assays for the ADGRE aGPCRs, and identification of soluble agonists that can induce G protein activation. Recombinant assays offer the means to identify better pharmacological tools to investigate and modulate biological functions, and to provide a starting point for the development of therapeutic agents. Our findings build on recent work, including the identification of activating peptides^12,15,48^, and synthetic ‘monobodies’ that activate GPR56^49^, providing optimism that functional probes for aGPCRs can be identified. There is now an opportunity to explore whether activating antibodies act to remove the NTF, or whether EMR2 (or another aGPCR) remains intact during the activation process. pAbs may have limited developability but mAbs, selected for their ability to activate G proteins, could elucidate aGPCRs signalling mechanisms on biologically relevant cells or tissues. Raising antibodies against similar regions of other aGPCRs may be a good strategy to identify such activators.

## Materials and Methods

### Gene and plasmid Information

The CD97 ‘full length’ clone (ADGRE5; Entrez gene ID: 976) was the splice variant encoding the signal sequence (MGGRV to GAETQ), EGF modules 1 (DSRGC to TETCD), 2 (DINEC to ENTCQ) and 5 (DVDEC to DTVEC), and the remainder of the coding region (DMTFS to SESGI). The truncated ‘CTF’ derivative was designed with a starting methionine codon followed by the sequence SSFAI, which follows the GPS cleavage site at lysine 437 in the splice variant used, continuing to the end of the coding sequence DTVEC. The EMR2 ‘full length’ clone (ADGRE2; Entrez gene ID: 30817) contained the natural signal sequence (MGGRV to GAETQ), all five EGF modules (DSRGC to DTVCE) and the remainder of the coding region (DMTFS to PSTVN). The CTF derivative of EMR2 was designed with a starting methionine codon followed by the sequence SSFAV, which follows the GPS cleavage site at lysine 517 in the splice variant used, continuing to the end of the coding sequence (PSTVN). For yeast episomal expression, CD97 and EMR2 coding sequences were amplified using polymerase chain reaction and sub-cloned into the *Hind*III site of pPGK (pDT-PGK)^50^ or p426GPD^51^. Open reading frames were preceded by the sequence AAAAAA. pPGK was the vector control in Fig. 1a,b. For chromosomal integration, an integrating vector (p306GPD) was created, in which a *Pvu*II fragment containing the GPD promoter, polylinker and *CYC1* terminator region from p426GPD was sub-cloned into the *Pvu*II-cut backbone of pRS306^52^. CD97 and EMR2 sequences were amplified using polymerase chain reaction and sub-cloned into the *Hind*III site of pRS306GPD. The resulting constructs were linearised using *Nsi*I and transformed into the respective MMY host strains.

For mammalian cell expression, the same EMR2 and CD97 sequences were used as for yeast expression. EMR2-FL was amplified by PCR and cloned by TA cloning (Invitrogen/Thermo Fisher, Waltham, MA, USA) into pCR3.1 (Invitrogen/Thermo Fisher); EMR2-CTF was taken as a *Hind*III fragment from p306GPD-EMR2 and sub-cloned into the *Hind*III site of pcDNA3.1(-) (Invitrogen/Thermo Fisher). CD97-FL was amplified with *Xho*I and *Not*I sites immediately flanking the open reading frame and cloned into pcDNA3. CD97-CTF was excised as a *Hind*III fragment from p306GPD-CD97-CTF and sub-cloned into the *Hind*III site of pcDNA3.1(-). pcDNA3/pcDNA3.1 (Invitrogen/Thermo Fisher) was the vector control. Human angiotensin AT1 gene (Entrez 185) was amplified with *Bam*HI sites immediately flanking the Open Reading Frame and cloned into the *Bam*HI site of pcDNA3.

For Bacmam construction, EMR2 was excised from pCR3.1 as a HindIII/NotI fragment and sub-cloned into pFastBacmam-1, a derivative of pFastBac1 (Invitrogen/Thermo Fisher), in which the polyhedrin promoter is replaced with a 3.1 kb NruI/Bst1107I fragment from pcDNA3 containing the CMV IE promoter, polylinker, BGH poly A site and the SV40 promoter driving expression of the G418 resistance gene.

The G_α16_ gene (Entrez gene ID 2769) containing 700 bp of upstream and 300 bp of downstream flanking sequence was sub-cloned into pCIH^53^. The mouse G_α15_ gene (Entrez gene ID 14676) was sub-cloned into pcDNA3.1. The G_16z49_ and G_16/13–49_ constructs contained G_α16_ with the C-terminal 49 amino acids replaced with those from G_αz_ and G_α13_ respectively, sub-cloned into vectors pFastNot (derived from pFastBacMam-1) and pcDNA3.1 respectively.

### Yeast strains

Yeast strains used were MMY12 (Gpa1), MMY14 (Gpa1/G_αq_), MMY15 (Gpa1/G_αs_), MMY16 (Gpa1/G_α16_), MMY19 (Gpa1/G_α12_), MMY20 (Gpa1/G_α13_), MMY21 (Gpa1/G_α14_), MMY22 (Gpa1/G_αo_), MMY23 (Gpa1/G_αi1_), MMY24 (Gpa1/G_αi3_), MMY25 (Gpa1/G_αz_), as described in Brown *et al.*^54^ and references therein; MMY15 is an alternative to the previously described strain MMY28^37^. Chromosomal integration of EMR2 and CD97 was used in strains MMY15, 16 and 20 due to toxic effects of episomal constructs in these strains, and controlled with integration of pRS306GPD; otherwise, episomal expression was used. The somatostatin receptor sst_2_ was chromosomally integrated into MMY16 to produce strain YIG90. Yeast transformations were carried out by the methods previously described^37^.

### Reagents and antibodies

All chemicals and reagents were purchased from Sigma-Aldrich (St. Louis, MO, USA) unless otherwise specified. The somatostatin peptide SRIF-14 (S9129) was dissolved in water. mAbs used for cell activation were 2A1 (EMR2-specific mAb, MCA2330; Bio-Rad AbD Serotec, Hercules, CA, USA) and mouse monoclonal IgG1 (Clone 11711; R&D Systems, Minneapolis, MN, USA). Polyclonal Sheep anti-Human EMR2 Antibody (pAb; clone AF4894) was purchased from R&D Systems.

### Cell culture, mammalian cell expression and transient transfection

HEK 293 T were cultured in DMEM/F-12 Media (Invitrogen/Thermo Fisher) supplemented with 10% Foetal Bovine Serum, 5 mM Glutamax and 5 mM HEPES (Gibco/Thermo Fisher). All cells were cultured at 37 °C in a 5% CO_2_ incubator.

HEK 293 T cells were transfected with plasmids (0.4 μg DNA/cm^2^ of culture vessel) using a GSK-proprietary transfection reagent. Transfected cultures were incubated at 37 °C and 5% CO_2_ for 48–72 hours. Co-transfections were performed with equal proportions of GPCR and G protein DNA. After transfection, cells were detached using a non-enzyme-based detachment solution (HBSS/EDTA, Gibco/Thermo Fisher) and plated for assay.

### Bacmam construction and transduction

BacMam is a modified baculovirus designed for transduction and expression in mammalian cells^55^. For virus generation, DH10bac cells (Invitrogen/Thermo Fisher) were transformed with plasmid pFastBacMam-EMR2. Virus was generated using the ‘Bac-to-Bac’ system (Invitrogen/Thermo Fisher) according to the manufacturer’s protocol. HEK 293 T cells were transduced with BacMam viruses encoding EMR2 at 10^6^ cells per transduction and multiplicity of infection of 100, in DMEM supplemented with 10% Foetal Bovine Serum and 0.1 mM non-essential amino acids. Following incubation at 37 °C overnight, medium was changed and cells incubated a further 24 h.

### Immunoprecipitation and western blotting

Cells were washed and incubated with agitation in cold lysis buffer (50 mM Tris-HCl pH 8.0, 1% NP-40, 150 mM NaCl, 0.1% deoxycholate, 1 mM EDTA) for 30 min and supernatants harvested, pre-cleared and incubated on ice overnight with anti-EMR2 R&D Systems AF4894 (sheep) 0.2 mg/ml or anti-human EMR2 Serotec MCA2330 monoclonal Ab clone 2A1 (mouse) 0.2 mg/ml. Mixtures were incubated with agarose beads for 1 h, beads were washed three times in 10 mM Tris-HCl pH 8.0, 0.1% NP-40 w/v, resuspended in 50 μl sample buffer and heated at 95 °C prior to electrophoresis on NuPAGE 4–12% Bis-tris gel (Invitrogen/Thermo Fisher), in NuPAGE MOPS SDS running buffer (NP0001) containing NuPAGE antioxidant (NP0005). Size marker was Rainbow Coloured Protein molecular weight RPN756, (GE Healthcare, Chicago, IL, USA).

Proteins were transferred onto nitrocellulose membrane. This was cut into two panels (as indicated in Fig. 6a,b). Each panel was incubated with appropriate dilutions of primary antibodies (Probe), in blocking buffer, overnight at 4 °C. Membranes were incubated with secondary antibodies: Anti-mouse 800 (Goat) (ref 926–31062 Li-COR Biosciences, Lincoln, NE, USA), anti-sheep Invitrogen Alexafluor-680 (donkey) A21102 in blocking buffer for 2 h, washed four times in PBS + 0.1% Tween 20. Images were acquired by scanning on Odyssey Imaging System (Li-COR Biosciences).

### Yeast assays

Yeast assays measure yeast cell growth enabled by the production of histidine from the *FUS1-HIS3* reporter, which is induced by the pheromone-response pathway following GPCR activation. Growth is quantified by addition of the fluorogenic reagent Fluorescein-Di-β-D-Glucopyranoside (FDGlu; Invitrogen/Thermo Fisher), which is converted to fluorescein, in proportion to cell number, by the action of secreted exoglucanase^37^. Assays were carried out as previously described^37^, in the presence of 1 mM 3-amino triazole and 10 μM FDGlu with a cell seeding density of 0.02 A_600_ Units in 384-well black walled, clear-bottomed plates (Nunc/Thermo Fisher). Plates were incubated for 24 hours at 30 °C and fluorescein product measured using a Tecan Spectrafluor Plus (Tecan, Mannedorf, Switzerland) plate reader (ex 485 nm/em 535 nm). Data were analysed using Prism version 5.0 (GraphPad Software, Inc., San Diego, CA, USA).

### Cell proliferation assay

HEK 293 T cells were plated at 70,000 cells/well in 6 well plates using basal cell culture media and transiently transfected using 0.4 μg/cm^2^ plasmid DNA. The cell plate was placed into the IncuCyte (Essen Instruments, Baraboo, WI, USA) apparatus inside a 37 °C, 5% CO_2_ cell culture incubator. Images of the cell monolayer confluence were recorded every 4 hours for a total duration of 72 hours.

### cAMP TR-FRET assay

HEK 293 T cells were seeded into T25 flasks at 60,000 cells/cm^2^ and incubated at 37 °C with 5% CO_2_ overnight. Cells were transiently transfected for 48 hours in total, for the last 16 hours of incubation 100 ng/ml pertussis toxin was present in the media. Constitutive levels of cAMP accumulation were measured using the homogeneous, Time-Resolved Fluorescence Resonance Energy Transfer (TR-FRET) LANCE cAMP kit (Perkin-Elmer, Waltham, MA, USA) as per manufacturer’s instructions. The assay was performed using 10,000 cells/well in white, low volume 384-well plates (Greiner Bio-One, Monroe, NC, USA) in the presence of 100 μM Rolipram and between 200–800 nM Forskolin pre-stimulation. The TR-FRET signal was read on a Viewlux imager (Perkin-Elmer) after a 3 hour room temperature incubation.

### NFAT-Luciferase reporter assay

HEK 293 T cells were seeded into T75 flasks at 90,000 cells/cm^2^ and incubated at 37 °C and 5% CO_2_ overnight. The following day, cells were transiently transfected for 48 hours in total; for the last 24 hours, cells were plated at 10,000 cells/well into sterile, white, 384-well plates (Nunc/Thermo Fisher Scientific) containing 5–10 mg/ml of EMR2 antibody or 25 μM AngII (human Angiotensin II, Sigma-Aldrich) in phenol-red free OptiMEM1 medium (Gibco/Thermo Fisher). Luminescence was measured using the Steady-Glo Luciferase Assay System (Promega, Madison, WI, USA) as per manufacturer’s instructions. The Luminescent signal was read on a EnVision imager (Perkin-Elmer).

### IP-one HTRF assay

HEK 293 T cells were seeded into T75 flasks at 90,000 cells/cm^2^ and incubated at 37 °C and 5% CO_2_ overnight. The following day cells were transiently transfected for 24 hours. IP_1_ accumulation was measured using Homogeneous Time-Resolved Fluorescence (HTRF) IP-ONE Tb Kit (Cisbio, Codolet, France) as per manufacturer’s instruction. The assay was perfomed using 10,000 cells/well into white, low volume 384-well plates (Greiner Bio-One) The HTRF signal was read on an Envision imager (Perkin-Elmer) after a 1 hour room temperature incubation.

## Supplementary information

Supplementary Information.

## References

[1] Hamann J (2015). International Union of Basic and Clinical Pharmacology. XCIV. Adhesion G protein-coupled receptors. Pharmacol. Rev..

[2] Arac D (2012). Dissecting signaling and functions of adhesion G protein-coupled receptors. Ann. N. Y. Acad. Sci..

[3] Liebscher Ines, Schöneberg Torsten, Prömel Simone (2013). Progress in demystification of adhesion G protein-coupled receptors. Biological Chemistry.

[4] Monk KR (2015). Adhesion G Protein-Coupled Receptors: From *In Vitro* Pharmacology to *In Vivo* Mechanisms. Mol. Pharmacol..

[5] Liebscher I (2014). New functions and signaling mechanisms for the class of adhesion G protein-coupled receptors. Ann. N. Y. Acad. Sci..

[6] Glenn TD, Talbot WS (2013). Analysis of GPR126 function defines distinct mechanisms controlling the initiation and maturation of myelin. Dev..

[7] Petersen SC (2015). The adhesion GPCR GPR126 has distinct, domain-dependent functions in Schwann cell development mediated by interaction with laminin-211. Neuron.

[8] Hamann J, Vogel B, van Schijndel GM, van Lier RA (1996). The seven-span transmembrane receptor CD97 has a cellular ligand (CD55, DAF). J. Exp. Med..

[9] Xu L, Begum S, Hearn JD, Hynes RO (2006). GPR56, an atypical G protein-coupled receptor, binds tissue transglutaminase, TG2, and inhibits melanoma tumor growth and metastasis. Proc. Natl. Acad. Sci. USA.

[10] Stacey Martin, Chang Gin-Wen, Davies John Q., Kwakkenbos Mark J., Sanderson Ralph D., Hamann Jörg, Gordon Siamon, Lin Hsi-Hsien (2003). The epidermal growth factor–like domains of the human EMR2 receptor mediate cell attachment through chondroitin sulfate glycosaminoglycans. Blood.

[11] Langenhan T, Aust G, Hamann J (2013). Sticky signaling–adhesion class G protein-coupled receptors take the stage. Sci. Signal..

[12] Stoveken HM, Hajduczok AG, Xu L, Tall GG (2015). Adhesion G protein-coupled receptors are activated by exposure of a cryptic tethered agonist. Proc. Natl. Acad. Sci. USA.

[13] Arac D (2012). A novel evolutionarily conserved domain of cell-adhesion GPCRs mediates autoproteolysis. EMBO J..

[14] Purcell RH, Hall RA, Adhesion G (2018). Protein-Coupled Receptors as Drug Targets. Annu. Rev. Pharmacol. Toxicol..

[15] Liebscher I (2014). A tethered agonist within the ectodomain activates the adhesion G protein-coupled receptors GPR126 and GPR133. Cell Rep..

[16] McKnight, A. J. & Gordon, S. EGF-TM7: a novel subfamily of seven-transmembrane-region leukocyte cell-surface molecules. *Immunol. Today* **17**, 283–287, doi:0167-5699(96)80546-9 [pii] (1996). 10.1016/0167-5699(96)80546-9 8962632

[17] Lin HH, Stacey M, Hamann J, Gordon S, McKnight AJ (2000). Human EMR2, a novel EGF-TM7 molecule on chromosome 19p13.1, is closely related to CD97. Genomics.

[18] Aust G, Hamann J, Schilling N, Wobus M (2003). Detection of alternatively spliced EMR2 mRNAs in colorectal tumor cell lines but rare expression of the molecule in colorectal adenocarcinomas. Virchows Arch..

[19] Gray JX (1996). CD97 is a processed, seven-transmembrane, heterodimeric receptor associated with inflammation. J. Immunol..

[20] Kop EN (2005). Identification of the epidermal growth factor-TM7 receptor EMR2 and its ligand dermatan sulfate in rheumatoid synovial tissue. Arthritis Rheum..

[21] Kwakkenbos MJ (2005). Expression of the largest CD97 and EMR2 isoforms on leukocytes facilitates a specific interaction with chondroitin sulfate on B cells. J. Leukoc. Biol..

[22] Eichler W (2000). CD97 isoform expression in leukocytes. J. Leukoc. Biol..

[23] Wandel E, Saalbach A, Sittig D, Gebhardt C, Aust G (2012). Thy-1 (CD90) is an interacting partner for CD97 on activated endothelial cells. J. Immunol..

[24] Wang T (2005). CD97, an adhesion receptor on inflammatory cells, stimulates angiogenesis through binding integrin counterreceptors on endothelial cells. Blood.

[25] Jaspars LH, Vos W, Aust G, Van Lier RA, Hamann J (2001). Tissue distribution of the human CD97 EGF-TM7 receptor. Tissue Antigens.

[26] Hamann J (2000). Molecular cloning and characterization of mouse CD97. Int. Immunol..

[27] Aust G (2002). CD97, but not its closely related EGF-TM7 family member EMR2, is expressed on gastric, pancreatic, and esophageal carcinomas. Am. J. Clin. Pathol..

[28] Kwakkenbos MJ (2002). The human EGF-TM7 family member EMR2 is a heterodimeric receptor expressed on myeloid cells. J. Leukoc. Biol..

[29] Chang GW (2007). CD312, the human adhesion-GPCR EMR2, is differentially expressed during differentiation, maturation, and activation of myeloid cells. Biochem. Biophys. Res. Commun..

[30] Boyden SE (2016). Vibratory Urticaria Associated with a Missense Variant in ADGRE2. N. Engl. J. Med..

[31] Ward Y (2011). LPA receptor heterodimerizes with CD97 to amplify LPA-initiated RHO-dependent signaling and invasion in prostate cancer cells. Cancer Res..

[32] Ward Y (2018). Platelets Promote Metastasis via Binding Tumor CD97 Leading to Bidirectional Signaling that Coordinates Transendothelial Migration. Cell Rep..

[33] Yin Y (2018). CD97 Promotes Tumor Aggressiveness Through the Traditional G Protein-Coupled Receptor-Mediated Signaling in Hepatocellular Carcinoma. Hepatology.

[34] Gupte J (2012). Signaling property study of adhesion G-protein-coupled receptors. FEBS Lett..

[35] Yona S (2008). Ligation of the adhesion-GPCR EMR2 regulates human neutrophil function. FASEB J..

[36] I, K. Y. *et al*. Activation of Adhesion GPCR EMR2/ADGRE2 Induces Macrophage Differentiation and Inflammatory Responses via Galpha16/Akt/MAPK/NF-kappaB Signaling Pathways. *Front Immunol* **8**, 373, 10.3389/fimmu.2017.00373 (2017). 10.3389/fimmu.2017.00373 PMC5376562 28421075

[37] Dowell SJ, Brown AJ (2009). Yeast assays for G protein-coupled receptors. Methods Mol. Biol..

[38] Brown Andrew J., Dyos Susan L., Whiteway Malcolm S., White Julia H. M., Watson Marie-Ange E. A., Marzioch Martina, Clare Jeff J., Cousens Diane J., Paddon Chris, Plumpton Chris, Romanos Mike A., Dowell Simon J. (2000). Functional coupling of mammalian receptors to the yeast mating pathway using novel yeast/mammalian G protein ?-subunit chimeras. Yeast.

[39] Katada T, Tamura M, Ui M (1983). The A protomer of islet-activating protein, pertussis toxin, as an active peptide catalyzing ADP-ribosylation of a membrane protein. Arch. Biochem. Biophys..

[40] Wu D, Katz A, Simon MI (1993). Activation of phospholipase C beta 2 by the alpha and beta gamma subunits of trimeric GTP-binding protein. Proc. Natl Acad. Sci. USA.

[41] Rao A, Luo C, Hogan PG (1997). Transcription factors of the NFAT family: regulation and function. Annu. Rev. Immunol..

[42] Park SJ (2013). Lysophosphatidylethanolamine utilizes LPA(1) and CD97 in MDA-MB-231 breast cancer cells. Cell Signal..

[43] Nakafuku M, Itoh H, Nakamura S, Kaziro Y (1987). Occurrence in Saccharomyces cerevisiae of a gene homologous to the cDNA coding for the alpha subunit of mammalian G proteins. Proc. Natl Acad. Sci. USA.

[44] Dietzel C, Kurjan J (1987). The yeast SCG1 gene: a G alpha-like protein implicated in the a- and alpha-factor response pathway. Cell.

[45] Matsuoka M, Itoh H, Kozasa T, Kaziro Y (1988). Sequence analysis of cDNA and genomic DNA for a putative pertussis toxin-insensitive guanine nucleotide-binding regulatory protein alpha subunit. Proc. Natl Acad. Sci. USA.

[46] Fong HK, Yoshimoto KK, Eversole-Cire P, Simon MI (1988). Identification of a GTP-binding protein alpha subunit that lacks an apparent ADP-ribosylation site for pertussis toxin. Proc. Natl Acad. Sci. USA.

[47] Hinton DR (1990). Novel localization of a G protein, Gz-alpha, in neurons of brain and retina. J. Neurosci..

[48] Schoneberg T, Liebscher I, Luo R, Monk KR, Piao X (2015). Tethered agonists: a new mechanism underlying adhesion G protein-coupled receptor activation. J. Recept. Signal. Transduct. Res..

[49] Salzman GS (2017). Stachel-independent modulation of GPR56/ADGRG1 signaling by synthetic ligands directed to its extracellular region. Proc. Natl Acad. Sci. USA.

[50] Kang YS, Kane J, Kurjan J, Stadel JM, Tipper DJ (1990). Effects of expression of mammalian G alpha and hybrid mammalian-yeast G alpha proteins on the yeast pheromone response signal transduction pathway. Mol. Cell Biol..

[51] Mumberg D, Muller R, Funk M (1994). Regulatable promoters of Saccharomyces cerevisiae: comparison of transcriptional activity and their use for heterologous expression. Nucleic Acids Res..

[52] Sikorski RS, Hieter P (1989). A system of shuttle vectors and yeast host strains designed for efficient manipulation of DNA in Saccharomyces cerevisiae. Genet..

[53] Rees S., Coote J., Stables J., Goodson S., Harris S., Lee M.G. (1996). Bicistronic Vector for the Creation of Stable Mammalian Cell Lines that Predisposes All Antibiotic-Resistant Cells to Express Recombinant Protein. BioTechniques.

[54] Brown AJ (2011). Pharmacology of GPR55 in yeast and identification of GSK494581A as a mixed-activity glycine transporter subtype 1 inhibitor and GPR55 agonist. J. Pharmacol. Exp. Ther..

[55] Pfohl JL (2002). Titration of KATP channel expression in mammalian cells utilizing recombinant baculovirus transduction. Receptors Channels.

