# Author Correction: G Protein-Coupling of Adhesion GPCRs ADGRE2/EMR2 and ADGRE5/CD97, and Activation of G Protein Signalling by an Anti-EMR2 Antibody

**DOI:** 10.1038/s41598-020-62011-0

**Published:** 2020-03-17

**Authors:** Nisha Bhudia, Sapna Desai, Natalie King, Nicolas Ancellin, Didier Grillot, Ashley A. Barnes, Simon J. Dowell

**Affiliations:** 1 Medicinal Science and Technology, GlaxoSmithKline, Stevenage, UK; 2 Excelya Clinical Research, Boulogne-Billancourt, France; 3 Oncodesign, Villebon-Sur-Yvette, France; 4 Censo Biotechnologies Ltd., Babraham, UK

Correction to: *Scientific Reports* 10.1038/s41598-020-57989-6, published online 22 January 2020

The Supplementary Information file that accompanies this Article contains errors in Supplementary Figure S1, where the figure keys are incorrect. The correct Figure S1 appears below as Figure 1.

**Figure 1:**
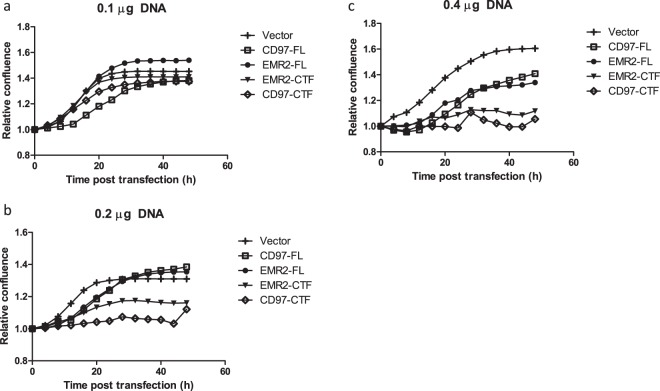
.

