# Supplementary material for: G Protein-Coupling of Adhesion GPCRs ADGRE2/EMR2 and ADGRE5/CD97, and Activation of G Protein Signalling by an Anti-EMR2 Antibody
Source: Sci Rep. 2020 Jan 22;10:1004. doi: 10.1038/s41598-020-57989-6 (PMC6976652; doi:10.1038/s41598-020-57989-6)
Supplement: Supplementary file 1 — Supplementary Information. [file 41598_2020_57989_MOESM1_ESM.pdf]

Supplementary Information

G Protein-Coupling of Adhesion GPCRs ADGRE2/EMR2 and ADGRE5/CD97, and Activation of G Protein Signalling by an Anti-EMR2 Antibody

Nisha Bhudia[1], Sapna Desai[1], Natalie King[2], Nicolas Ancellin[3], Didier Grillot[3], Ashley A. Barnes[4] and Simon J. Dowell[1*]

[1]Medicinal Science and Technology, GlaxoSmithKline, Stevenage, UK; [2]Excelya Clinical Research, Boulogne-Billancourt, France; [3]Oncodesign, Villebon-Sur-Yvette, France; [4]Censo Biotechnologies Ltd, Babraham, UK

*Corresponding author

Contact details:

Nisha Bhudia (nisha.6.bhudia@gsk.com)

Sapna Desai (sapna.x.desai@gsk.com)

Natalie King (eilatan90@gmail.com)

Nicolas Ancellin (nicolas.ancellin@oncodesign.com)

Didier Grillot (didier.grillot@oncodesign.com)

Ashley Barnes (ashley.barnes@censobio.com)

Simon Dowell (simon.j.dowell@gsk.com) – corresponding author

Figure S1

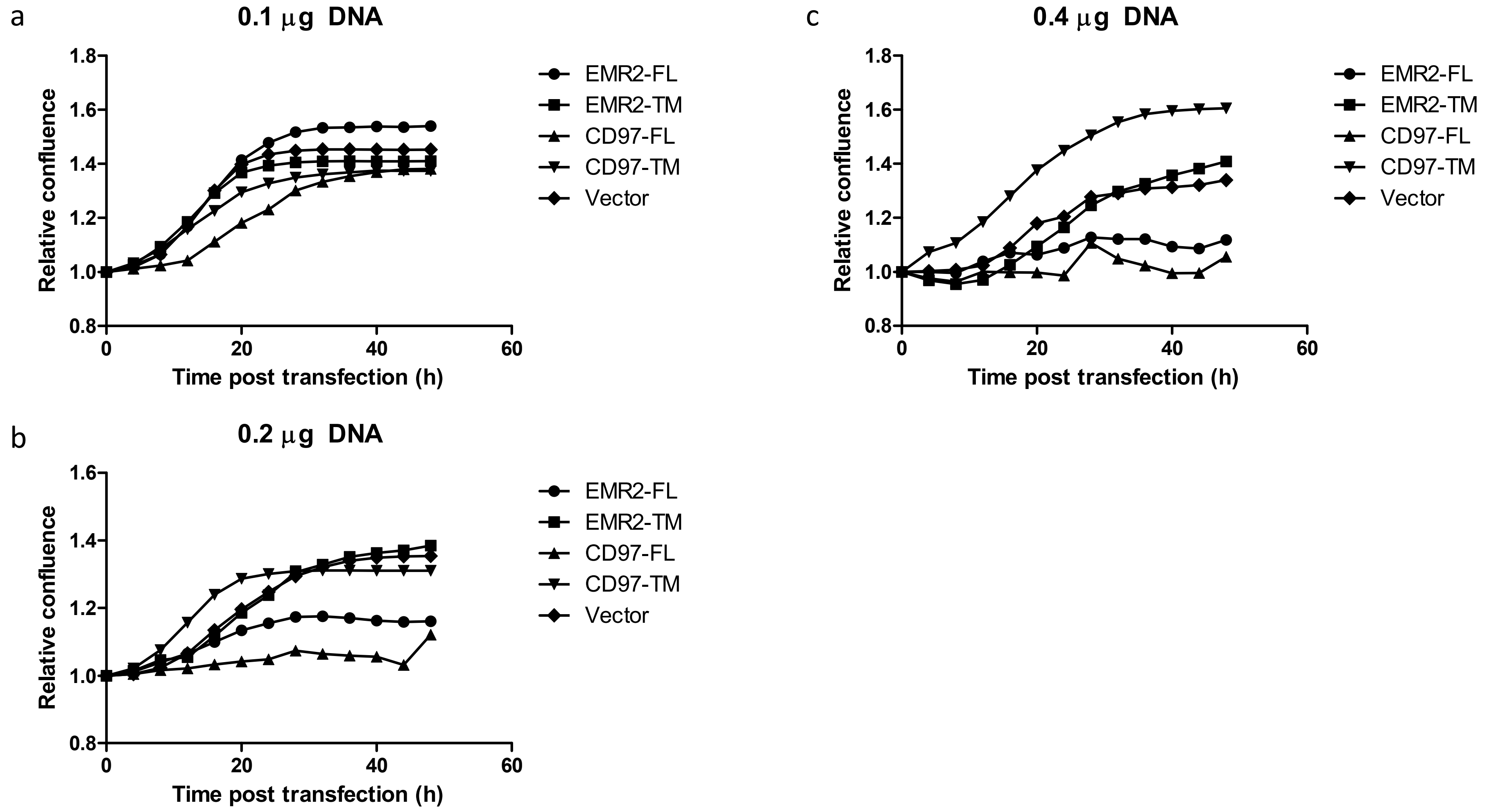

a
0.1 μg DNA
Relative confluence
Time post transfection (h)
EMR2-FL
EMR2-TM
CD97-FL
CD97-TM
Vector
c
0.4 μg DNA
b
0.2 μg DNA

Figure S2

a b

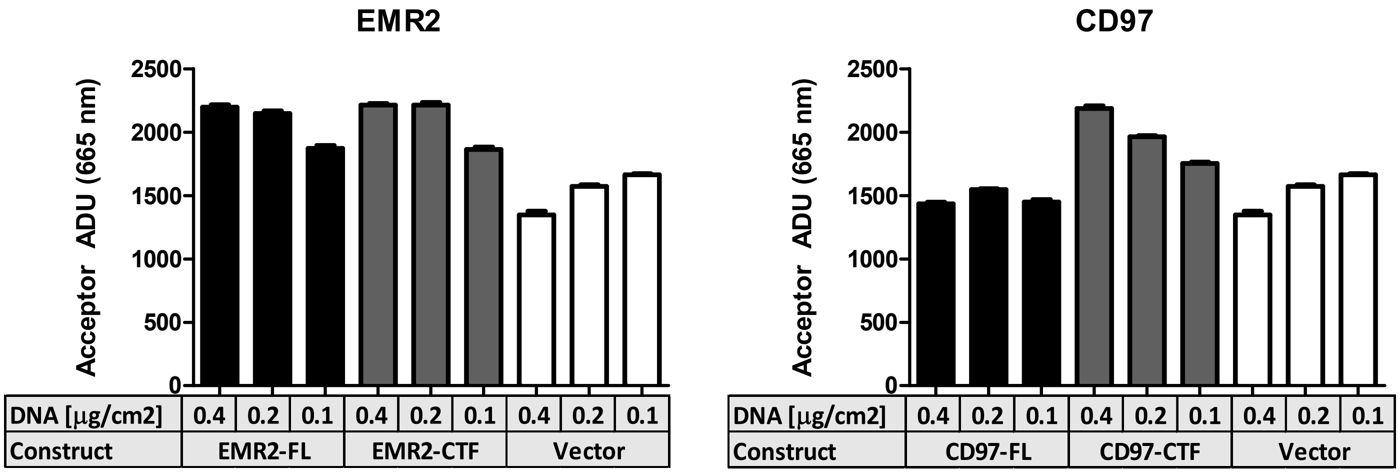
EMR2
Acceptor ADU (665 nm)
2500
2000
1500
1000
500
0
DNA [µg/cm2] 0.4 0.2 0.1 0.4 0.2 0.1 0.4 0.2 0.1
Construct EMR2-FL EMR2-CTF Vector
CD97
Acceptor ADU (665 nm)
2500
2000
1500
1000
500
0
DNA [µg/cm2] 0.4 0.2 0.1 0.4 0.2 0.1 0.4 0.2 0.1
Construct CD97-FL CD97-CTF Vector

Figure S3

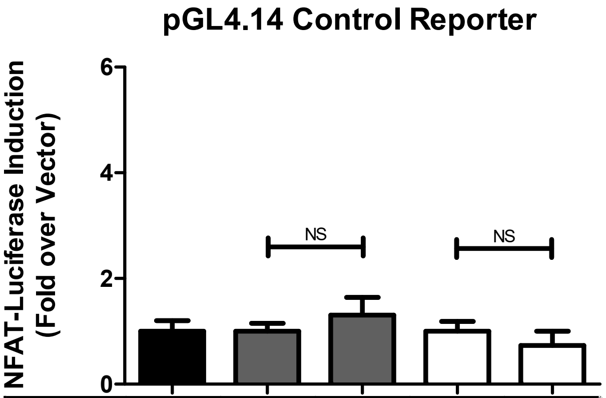

Vector	+	-	-	-	-
AT-1	-	+	+	-	-
AngII	-	-	+	-	-
EMR2-FL	-	-	-	+	-
EMR2-CTF	-	-	-	-	+
Gα16	-	-	-	+	+
